# TGF-β Is Required for Vascular Barrier Function, Endothelial Survival and Homeostasis of the Adult Microvasculature

**DOI:** 10.1371/journal.pone.0005149

**Published:** 2009-04-02

**Authors:** Tony E. Walshe, Magali Saint-Geniez, Arindel S. R. Maharaj, Eiichi Sekiyama, Angel E. Maldonado, Patricia A. D'Amore

**Affiliations:** Departments of Ophthalmology and Pathology, Harvard Medical School, Schepens Eye Research Institute, Boston, Massachusetts, United States of America; Helmholtz Zentrum München /Ludwig-Maximilians-University Munich, Germany

## Abstract

Pericyte-endothelial cell (EC) interactions are critical to both vascular development and vessel stability. We have previously shown that TGF-β signaling between EC and mural cells participates in vessel stabilization in vitro. We therefore investigated the role of TGF-β signaling in maintaining microvessel structure and function in the adult mouse retinal microvasculature. TGF-β signaling was inhibited by systemic expression of soluble endoglin (sEng) and inhibition was demonstrated by reduced phospho-smad2 in the adult retina. Blockade of TGF-β signaling led to increased vascular and neural cell apoptosis in the retina, which was associated with decreased retinal function, as measured by electroretinogram (ERG). Perfusion of the inner retinal vasculature was impaired and was accompanied by defective autoregulation and loss of capillary integrity. Fundus angiography and Evans blue permeability assay revealed a breakdown of the blood-retinal-barrier that was characterized by decreased association between the tight junction proteins zo-1 and occludin. Inhibition of TGF-β signaling in cocultures of EC and 10T1/2 cells corroborated the in vivo findings, with impaired EC barrier function, dissociation of EC from 10T1/2 cells, and endothelial cell death, supporting the role of EC-mesenchymal interactions in TGF-β signaling. These results implicate constitutive TGF-β signaling in maintaining the integrity and function of the adult microvasculature and shed light on the potential role of TGF-β signaling in vasoproliferative and vascular degenerative retinal diseases.

## Introduction

Formation, stabilization and specialization of the vasculature is a complex process that requires the coordinated action of a number of growth factors and a variety of heterotypic cellular interactions. Transforming growth factor-β1 (TGF-β1) is a multifunctional growth factor that is a well-established modulator of vascular cells [1]. In vitro studies indicate that TGF-β1 is activated upon contact between endothelial and mesenchymal cells [2] and that it mediates a variety of actions associated with vessel maturation including, inhibition of EC proliferation and migration, induction of pericyte differentiation, and production of basement membrane [2]–[4]. These observations suggest that local activation of TGF-β1 in vivo may be critical to vessel remodeling and stability.

The retinal microvasculature, the site of the inner blood retinal barriers, is one of the most stable microvascular beds in the body with EC turnover rates estimated in years [5]. Pericytes envelop EC tubes and are present at different pericyte-EC ratios depending upon the microvessel bed [6], [7]. Trypsin digests of the retinal vasculature have revealed a ratio of pericytes to ECs roughly equaled to 1, whereas ECs outnumber pericytes in other microvascular beds by as much as 10 to 1 [6]. In vitro studies demonstrate that contact between ECs and pericytes or astrocytes leads to TGF-β1 activation, a major determinant of TGF-β1 availability and signaling [8]. Moreover, the loss of retinal pericytes has been speculated to be permissive for the progression of diabetic retinopathy [9]. Taken together, these observations have led us to speculate that the high number of pericytes in the retina reflects a significant role for constitutive TGF-β1 signaling in maintenance of retinal microvascular integrity.

Binding of TGF-β1 dimers to TGFβ-receptor II (TGFβRII) leads to the recruitment of TGFβ-receptor I (TGFβRI), the formation of a tetrameric complex, phosphorylation and conformational changes in the intracellular domain of TGFβRI, and downstream activation of smad transcription factors. Most cell types express only one TGFβRI receptor, ALK5 [10]. In ECs, TGF-β1 activation of ALK5 is growth inhibitory and is thought to mediate vessel stability [10]. ECs also express the TGFβRI receptor ALK1, as well as the TGF-β1 co-receptor endoglin (also referred to as TGFβRIII). In contrast to ALK5 signaling, TGF-β1 signaling via endoglin or ALK-1 on ECs is associated with vessel destabilization, EC proliferation and migration, by limiting TGF-β1-ALK5 EC signaling [11]. Consistent with these findings, increased endoglin is a defining feature of proliferating vessels in tumors and is a current target for anti-cancer treatments (http://www.clinicaltrials.gov/ct2/show/NCT00582985?term=cd105&rank=1) [12].

The phenotypes of mice deficient in TGF-β1 and of naturally occurring mutations of TGF-β1 pathway support a role for TGF-β1 in formation and maintenance of the vasculature. Targeted deletion of *alk1, alk5, TGFβRII, endoglin* and *smad5* are all embryonic lethal, each with comparable cardiovascular defects, with some subtle differences [13]. *TGFβRII* null mice die around mid-gestation from defective yolk sac vascularization and hematopoiesis [14], whereas mice deficient in *TGFβRI* have defective yolk sac vasculogenesis, but normal hematopoiesis [15]. In humans, heterozygous mutations of either *endoglin* or *alk1* cause hereditary hemorrhagic telangiectasia (HHT)-1 or HHT-2, respectively, both characterized by vascular anomalies such as dilated vessels, edema, arterio-venous malformations, and pulmonary, liver and neurological problems due to vascular defects [16].

Systemic inhibition of TGF-β and VEGF, as a result of high levels of circulating placental derived soluble endoglin (sEng) and soluble fms-like tyrosine kinase 1 (sFlt1), respectively, have been reported to be involved in the pathogenesis of preeclampsia [17]. Preeclampsia is a condition of pregnancy characterized by systemic endothelial dysfunction, multiple end-organ ischemia, hypertension and proteinuria - a phenotype that is largely recapitulated by systemic inhibition of TGF-β and VEGF in pregnant rats [17]. Additionally, preeclampsia is associated with increased vascular permeability [18].

One consequence of reduced circulating TGF-β and VEGF is a decrease in endothelial formation of nitric oxide (NO) [17], a potent vasoactive molecule with anti-thrombogenic effects. Recent evidence demonstrates that administration of TGF-β1 can induce arteriogenesis in the peripheral circulation [19], however the role of endogenous TGF-β1 signaling in the quiescent adult microvasculature has not been defined. Therefore, we have examined the effects of systemic TGF-β inhibition on perfusion, permeability and function of adult microvasculature, using the retinal microvasculature as an easily accessible and clinically relevant model. The unique structure of the retina enables structural and functional analysis of microvessels in vivo, which are not feasible with other microvessel beds. In addition, we used an in vitro coculture system to identify the effects of TGF-β1 blockade on ECs and 10T1/2 cells (as mesenchymal precursors of pericytes) [20].

## Results

### Systemic inhibition of TGF-β decreases smad2 phosphorylation in the retina

**Figure 1:**
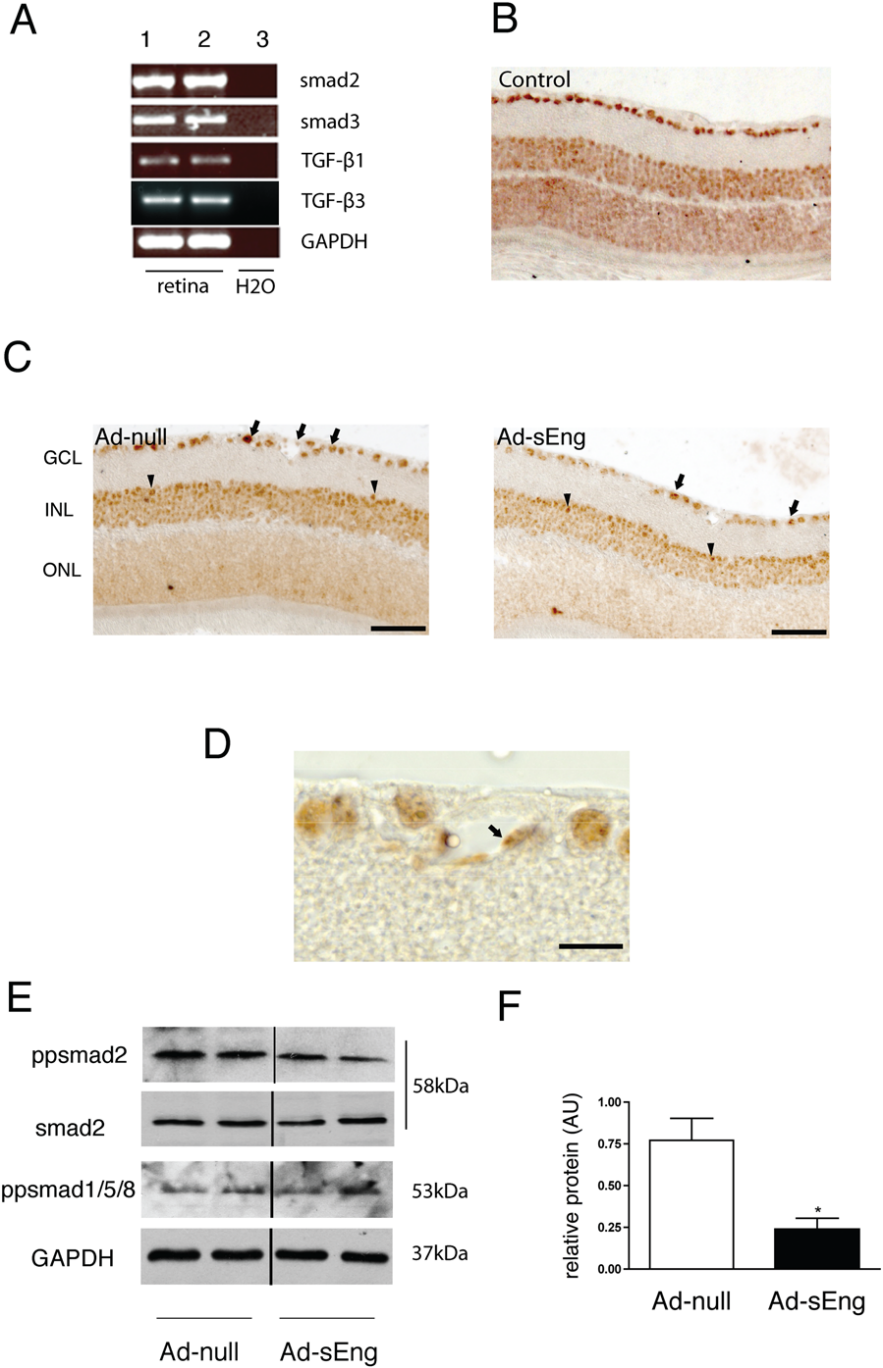
Systemic inhibition of TGF-β decreases retinal smad2 phosphorylation *in vivo*. (A) RT-PCR of adult murine retinal cDNA revealed the presence of smad2, smad3, TGF-β1 and TGF-β3. Lanes one and two are two retinal cDNA preparations. Lane three is a negative control with no RT enzyme. Immunohistochemical localization of pp-smad2 in retinas from (B) control (non infected animals) and (C) Ad-null and Ad-sEng-expressing mice (day 7) revealed pp-smad2 staining in nuclei of the ganglion cell layer (arrows) and the INL (arrowheads). sEng expression led to a decrease in the intensity of pp-smad2 staining. Scale = 50 µm. (D) Higher magnification of ppsmad2 staining in control mice, demonstrating nuclear pp-smad2 in retinal ECs (arrow). Scale = 20 µm. (E) Western blot analysis of pp-smad2, smad2, pp-smad1/5/8 and GAPDH in mouse retinas from Ad-null and Ad-sEng-expressing mice (day 7). sEng-expressing mice displayed a decrease in retinal pp-smad2 (F). Each lane corresponds to a protein preparation from one mouse retina. Quantification of ppsmad2 (n = 4, * p<0.05).

RT-PCR of cDNA collected from adult mouse retinas revealed the expression of smad2, smad3, TGF-β1 and TGF-β3 (Figure 1A). To evaluate the role of endogenous TGF-β signaling in vivo, adenovirus of Ad-sEng was used to systemically inhibit TGF-β signaling in mice; control mice included non-infected mice (Figure 1B) and mice infected with an Ad-null virus (Figure 1C). sEng binds both TGF-β1 and TGF-β3. In the retina, TGF-β1 is associated with endothelial, mural and microglial cells, whereas TGF-β3 is expressed mainly by ganglion and microglial cells [21]–[23]. One of the earliest events of TGF-β signal activation is phosphorylation of smad2 and its translocation to the nucleus [24]. We used immunohistochemistry for pp-smad2 to localize the cell types with active TGF-β signaling in the retina. In both control and treated mice, nuclear pp-smad2 was evident in the neurons of the ganglion cell layer (Figure 1C; arrows); the inner nuclear layer (INL), which contains bipolar, amacrine and horizontal cells (arrowheads); and, EC and mural cells (Figure 1D). The staining intensity of pp-smad2 was reproducibly reduced in Ad-sEng-expressing mice as compared to control mice (Figure 1C). Western blot analysis for pp-smad2 on whole retina lysates indicated a marked reduction of smad2 phosphorylation in Ad-sEng mice compared to the control after seven days (Figure 1E; Figure 1F quantification), demonstrating that systemic expression of sEng effectively neutralizes TGF-β signaling. No changes in total smad2 or phosphorylation of smad1/5/8 were noted after seven days of TGF-β neutralization (Figure 1E).

### Inhibition of TGF-β decreases retinal perfusion and impairs peripheral vascular autoregulation

**Figure 2:**
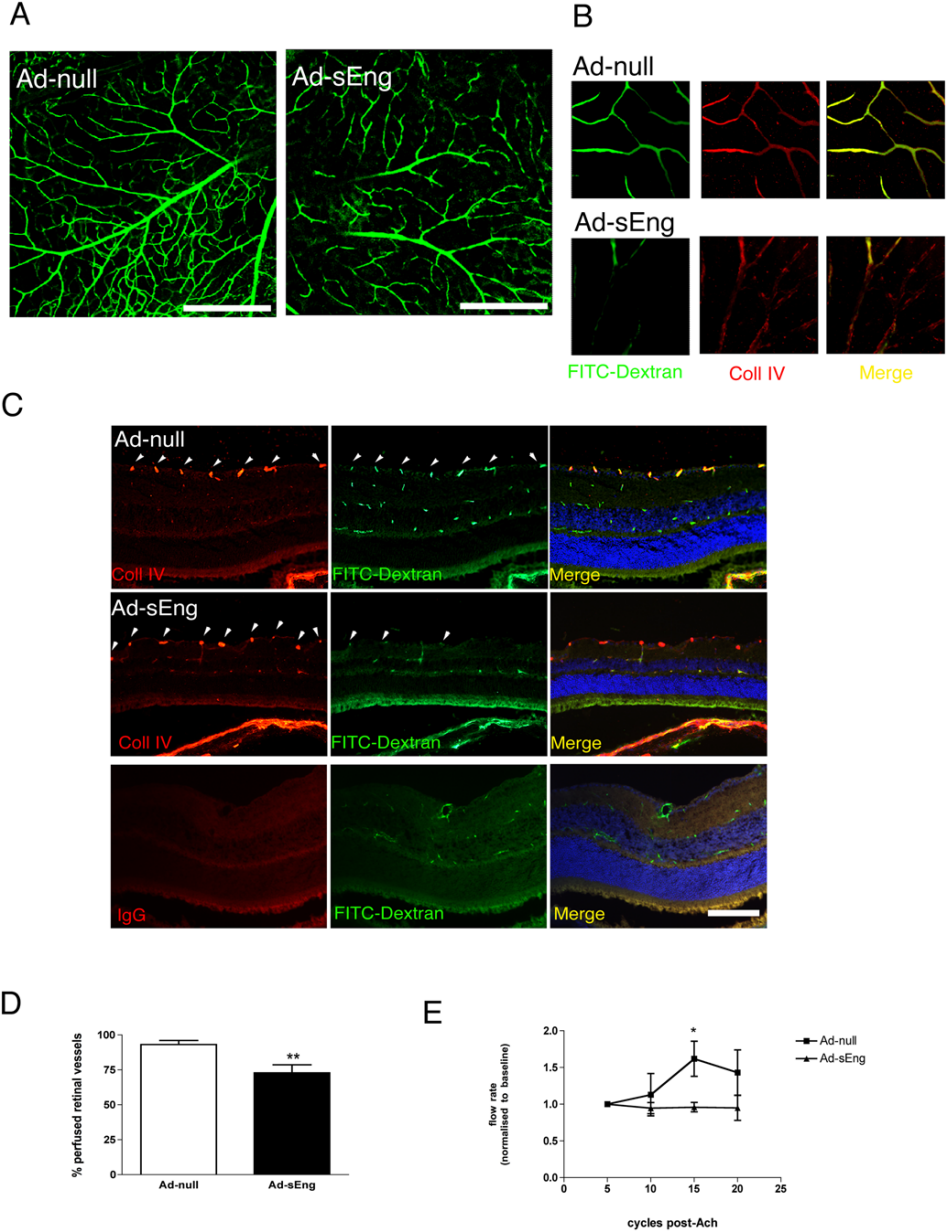
Inhibition of TGF-β decreases retinal perfusion and vascular autoregulation. After fourteen days of Ad-sEng and Ad-null expression, mice were injected with h.m.w. FITC-dextran through the left ventricle to localize perfused vessels. (A) Confocal analysis of retinal flat-mounts revealed reduced perfusion of the retina in the sEng-expressing mice compared to the control (representative photo of n = 12 mice). Scale bar = 200 µm. (B) The perfused vessels were visualized on retinal flat mounts by comparing the co-localization of type IV collagen (Cy3-red) and FITC and quantified on cryosections by comparing the number of vessels in the innermost vascular plexus (arrowheads) positive for both type IV collagen- and FITC to the number of vessels positive for type IV collagen but negative for FITC (C). (D) The retinas of sEng expressing mice show a marked reduction in the number of perfused vessels (n = 5, ** p<0.01). (E) After seven days of adenoviral expression, blood flow rates in the tail were measured non-invasively in response to intravascular injection of ACh. Measurements were made over 5 cycles pre-injection of ACh, normalized to 1 for each animal, and averaged at 5-cycle intervals post ACh injection. In Ad-null control mice, ACh increased tail vein blood flow rates 6–10 cycles post-injection, whereas blood flow rates were unchanged in Ad-sEng expressing mice (Ad-null: 1.619 µl/cycle, n = 5; Ad-sEng: 0.960 µl/cycle, n = 4, * p<0.01). Injection of 100 µl of saline in Ad-null or Ad-sEng mice elicited no response.

TGF-β1 regulates endothelial synthesis of numerous vasoactive agents such as NO and endothelin-1 [25]. To examine the effect of TGF-β1 blockade on microvascular perfusion, mice were perfused with high molecular weight FITC-dextran 14 days post adenovirus infection. Abnormal perfusion was apparent in retinal flat-mount preparations from sEng-expressing mice (Figure 2A), and co-staining for type IV collagen, a component of the capillary basement membrane, confirmed a lack of perfusion in some vessels of sEng expressing mice (Figure 2B). Microvascular perfusion was quantified on serial cryosections of dextran-perfused retinas. Though there was no change in the density of type IV collagen-positive vessels in the retinas of sEng-expressing mice compared to control mice, numerous of the type IV collagen-positive were FITC-dextran negative (Figure 2C), indicating non-perfusion. Quantification of the collagen and dextran-positive vessels in the inner layers of the retina confirmed a significant (approx. 25%) reduction in vascular perfusion in sEng-expressing mice (Figure 2D).

The retinal microvasculature maintains perfusion by autoregulation, where endothelial-derived NO induces mural cell relaxation thereby increasing local blood flow [26]. As altered peripheral blood flow is often used as a surrogate for retinal microvascular dysfunction [27], we monitored blood flow rates in the tail vasculature. Acetylcholine (ACh), an endothelium-dependent vasodilator, led to increased blood flow in control mice but not in sEng-expressing mice, indicating impaired endothelial vasoactive capacity (Figure 2E).

### TGF-β inhibition causes reduced endothelial barrier function in vivo and in vitro

**Figure 3:**
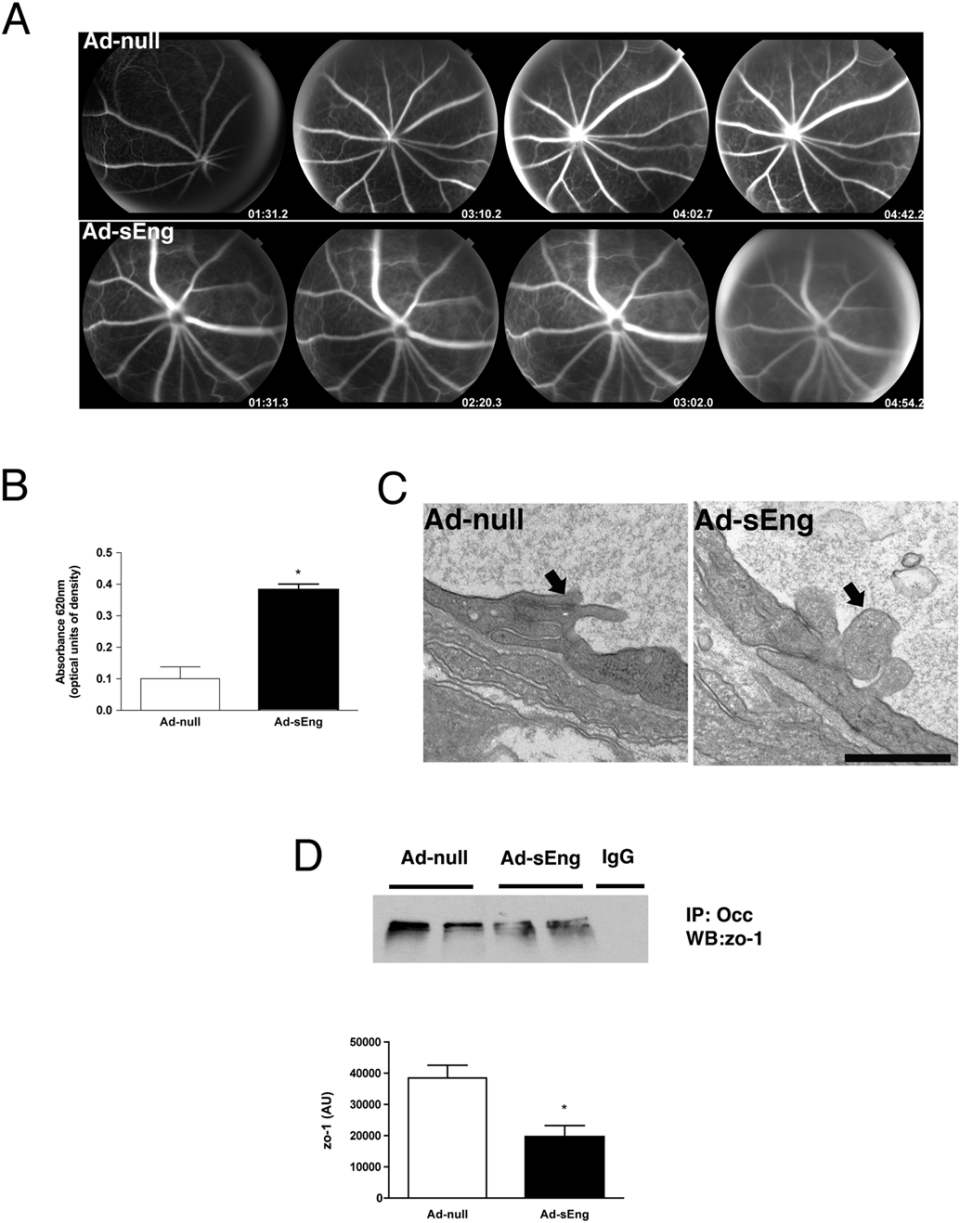
Inhibition of TGF-β decreases endothelial barrier function in vivo. (A) Fluorescein angiography of the retina was performed seven days following adenovirus injection. Ad-sEng-expressing mice displayed increased retinal permeability compared to control mice. The increased fluorescence leakage into the vitreous was observable at two min. Representative photographs are shown (n = 4). (B) Experimental mice infected for 14 days were injected intravenously with 2% Evans blue in PBS, followed by systemic PBS perfusion 40 min later. The eyes were removed, retinas dissected and quantified for Evans blue/albumin leakage. Inhibition of TGF-β resulted in a significant increase in the leakage of Evans blue into the extravascular space (n = 5, * p<0.05). (C) TEM micrographs of the inner retinal vasculature revealing tight junctions of Ad-null and sEng-expressing mice (day 14). sEng mice displayed alterations in tight junction structure, with disturbed interdigitation between adjacent ECs (arrow), a reduction in condensed regions characteristic of tight junctions and appearance of cellular debris between adjacent ECs (arrow). Scale = 0.5 µm. (D) Immunoprecipitation of occludin and western blotting for zo-1 demonstrated a significant decrease in association between occludin and zo-1 in lysates prepared from retinas of sEng-expressing mice (day 7). Each lane corresponds to an individual mouse (n = 4, * p<0.05).

Similar to the blood-brain-barrier, the retinal vasculature forms the blood-retinal barrier (BRB), which protects neural retina from neurotransmitters and potentially damaging circulating factors. Barrier function in vivo was examined by fundus angiography. Whereas vessels in Ad-null control mice were well-defined and did not exhibit evidence of fluorescein leakage, vessels of sEng-expressing mice appeared more diffuse due to a more rapid leakage of the dye (Figure 3A). In parallel experiments, extravasated Evans blue was quantified and confirmed a breakdown of the blood-retinal barrier in sEng-expressing mice (Figure 3B).

Barrier function is mediated by tight and adherens barrier proteins between adjacent ECs. Transmission electron microscopy (TEM) analysis of the superficial vascular plexus in sEng-expressing mice revealed structural alterations in tight junctions between microvascular ECs (Figure 3C). The tight junction proteins zo-1 and occludin constitute a major aspect of the blood retinal barrier [28]. Immunoprecipitation of occludin, followed by western blotting for zo-1, revealed a decrease in association between these two proteins in sEng-expressing mice (Figure 3D).

**Figure 4:**
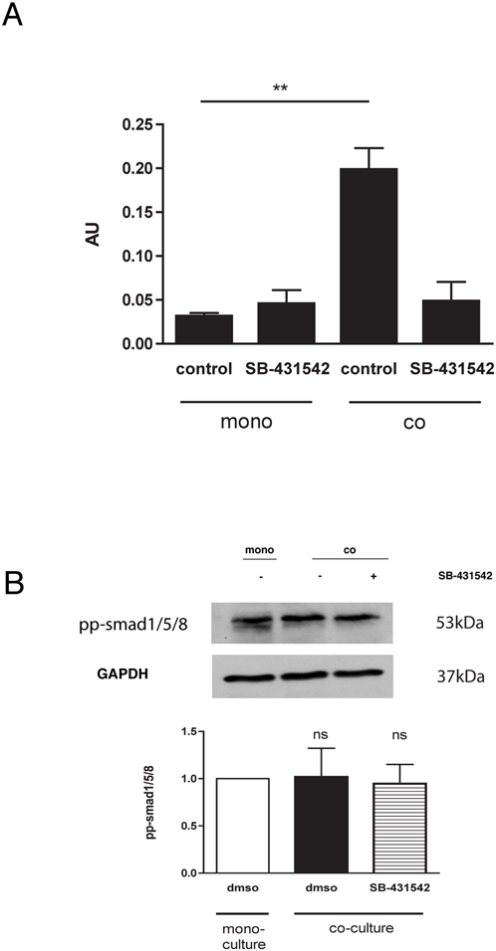
Co-culture of endothelial/mesenchymal cells activates endothelial smad2/3 signaling. (A) Transient transfection of ECs with CAGA-Luc smad2/3 reporter revealed that co-culture with 10T1/2 cells activated smad2/3 signaling. Pharmacological inhibition of TGF-β with 10 µM SB-431542 reversed this activation. (B) Co-culture of ECs with 10T1/2 cells did not alter phosphorylation of EC smad1/5/8.

To further define the function of TGF-β1 in barrier function, we utilized Transwell cocultures of EC and 10T1/2 cells (as undifferentiated mesenchymal pericyte precursors), which mimic the in vivo interaction between EC and mural cells and which we have shown leads to local activation of TGF-β1 [20]. Using a smad2/3 luciferase reporter construct (CAGA-Luc), we demonstrated activation of smad2/3 signaling in ECs in co-culture with 10T1/2 cells compared to EC mono-cultures (Figure 4A). Addition of SB-431542, a pharmacological inhibitor of TGFβRI /ALK5, reversed the co-culture effect of co-culture, but did not significantly alter baseline smad2/3 luciferase in EC mono-cultures or phosphorylation of smad1/5/8, downstream transcription factors of the TGF-βR1 – ALK1 (Figure 4B).

**Figure 5:**
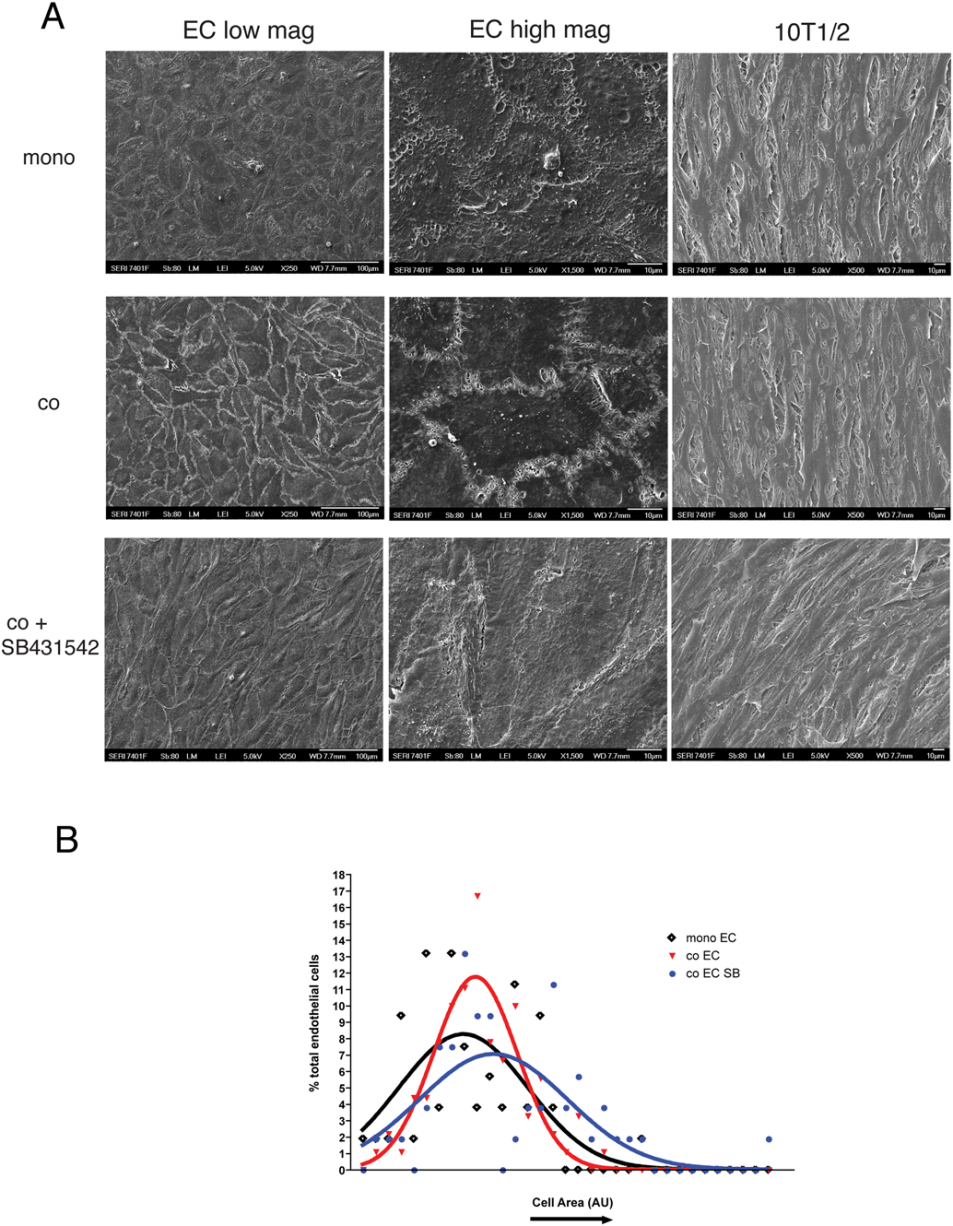
Inhibition of TGF-β decreases endothelial barrier function in vitro. (A) SEM analysis demonstrated a characteristic endothelial cobblestone morphology in EC monocultures. Co-culture enhanced the association between adjacent cells, which was reversed with addition of SB-431542. Scale bars = 100 µm (left panel); 10 µm (right panel). Cell size was calculated by tracing individual ECs using ImageJ. Morphology of 10T1/2 cells from cocultures with ECs was not visibly altered when compared to 10T1/2 monoculture or with the addition of SB431542. (B) Analysis of EC size in monoculture or coculture with 10T1/2 cells (+/−SB431542). Coculture did not significantly alter average cell size, however, EC size were more uniform when compared to EC mono-cultures. Addition of SB431542 led to larger and more heterogenous cell size.

Scanning electron microscopy (SEM) revealed enhanced association between adjacent ECs co-cultured with 10T1/2 cells (Figure 5A) and the size of EC in the co-cultures was more uniform than that of EC cultured alone (Figure 5B). The role of TGF-β1 signaling was assessed using SB-431542. Addition of SB-431542 to co-cultures reversed the co-culture-induced effects on interendothelial association (Figure 5A) and led to a reversion to heterogeneous EC cell sizes (Figure 5B). In contrast to changes in EC shape that were evident in co-culture with 10T1/2's, SEM analysis of 10T1/2's morphology revealed no obvious difference in their morphology whether in mono-culture, co-culture, or in the presence of SB-431542 (Figure 5A).

**Figure 6:**
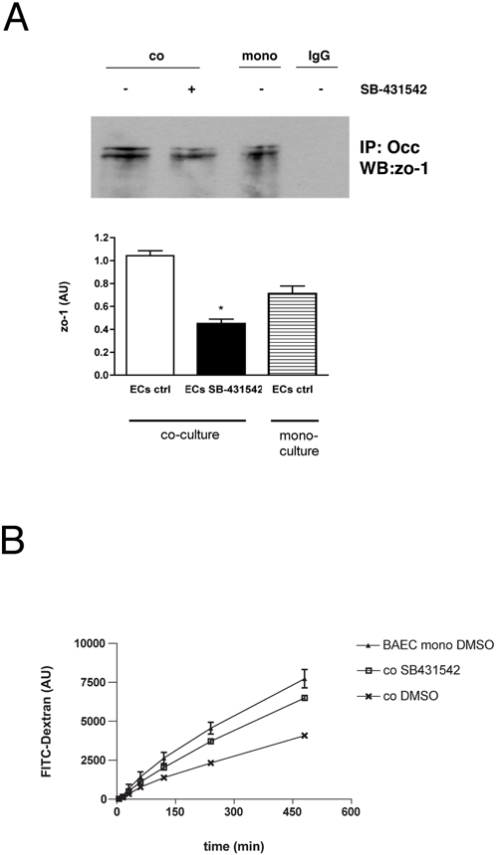
Inhibition of TGF-β impairs endothelial tight junction protein association. (A) Assay of tight junction components in Transwell cocultures of EC and 10T1/2 cells. Immunoprecipitation of occludin and western blotting for zo-1 revealed a significant increase in association between occludin and zo-1 in coculture compared to monocultures (* p<0.05), which was reversed by the addition of SB-431542. Non-specific IgG was used as a control for IP. (B) Assay of permeability in Transwell cocultures. Coculture of BAECs with 10T1/2 cells increased EC barrier function as measured by decreased flux of FITC-dextran from the upper to lower chamber. Addition of SB-431542 increased permeability of BAEC/10T1/2 cocultures.

Immunoprecipitation of occludin followed by western blotting for zo-1 revealed that coculture of EC with 10T1/2 cells led to an increased association between occludin and zo-1 in EC, which was significantly reduced by TGF-β1 inhibition (Figure 6A). Measurement of barrier function of EC in Transwell by the permeability of the EC layer to 40-kDa FITC-conjugated dextran [28] revealed that coculture of ECs with the 10T1/2 cells enhanced EC barrier function compared to that of ECs alone (Figure 6B). The reduction in permeability by the ALK5 inhibitor SB-431542 further implicates ALK5 signaling in the maintenance of EC barrier function.

### Effect of TGF-β inhibition on the ultrastructure of the retinal vasculature

**Figure 7:**
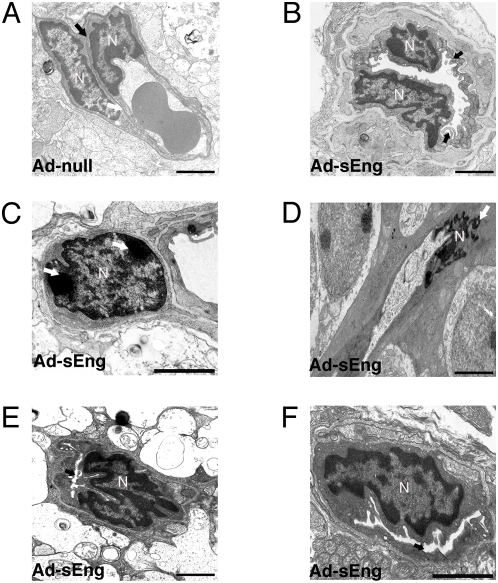
Ultrastructure of retinal vasculature following inhibition of TGF-β. (A) TEM micrograph of a microvessel in the ganglion cell layer from a retina of a control mouse expressing Ad-null (day 14). Nuclei of an EC and pericyte are apparent, with a defined basement membrane (arrow). (B)–(F) TEM micrographs of the retinal vessels from mice expressing Ad-sEng. (B) The lining of some ECs appeared ‘ruffled’ with finger-like processes protruding into the luminal space and multiple vacuoles within the cytoplasmic space (arrow). Nuclear condensation characteristic of apoptosis was apparent in some (C) pericytes and (D) ECs (arrows). (E) (F) Numerous vessels in the inner retinal layers displayed significant reductions in luminal diameter (arrows). (A)–(F) Scale = 2 µm.

TEM of capillaries in the ganglion cell layer of mice expressing Ad-null revealed normal ultrastructure, with close apposition between ECs and pericytes, a defined extracellular space, and few vacuoles (Figure 7A). In contrast, retinal microvessels of sEng-expressing mice were characterized by multiple abnormalities, particularly in the superficial vascular plexus. The luminal surface of some ECs exhibited a ruffled appearance, with luminal projections and increased vacuoles (Figure 7B). Nuclear condensation associated with apoptosis was apparent in some, but not all pericytes (Figure 7C) and ECs (Figure D), and, consistent with our findings of non-perfusion and impaired NO formation in sEng expressing mice, many vessels appeared completely or partially collapsed (Figures 7E and 7F).

### TGF-β inhibition leads to morphological and functional changes in neural retina

**Figure 8:**
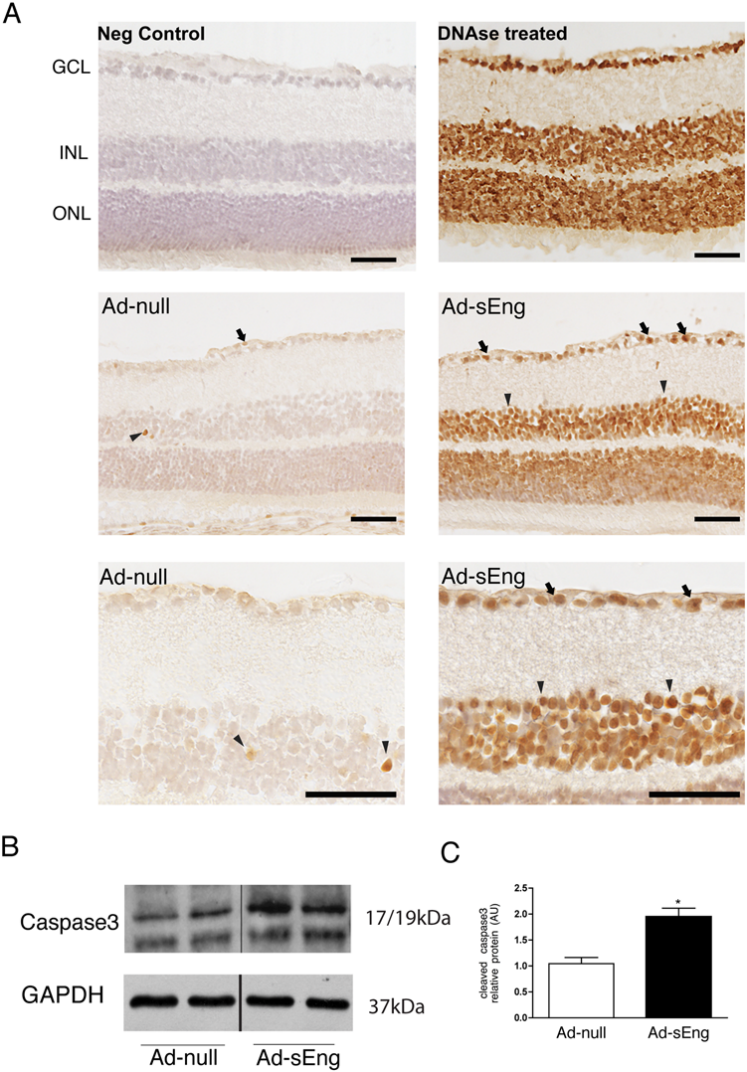
Inhibition of TGF-β increases retinal cell apoptosis. (A) Following 14 days of infection with Ad-null or Ad-sEng, retinas were examined for apoptotic cells via TUNEL staining. Retinas of sEng-expressing mice displayed a significant increase in the number of apoptotic cells. In the control retinas, occasional cells of the inner nuclear layer (INL) could be marked as apoptotic (arrowheads). Positive control tissue sections were treated with DNAse enzyme. Retinal sections were lightly counter-stained with hematoxylin QS to reveal nuclei (blue). Expression of sEng led to the induction of apoptosis of most of the cells in both the ganglion cell layer (arrows) and in the INL (arrowheads). Scale = 100 µm. (B) Lysates of whole retinas were analyzed via western blotting for cleaved caspase 3 levels. Inhibition of TGF-β led to increased cleaved caspase 3 as compared to control mice. (C) Quantification of (B) (* p<0.05).

TUNEL staining revealed an increase in the number of apoptotic cells in the ganglion cells layer, the inner, and outer nuclear layers of the retina (Figure 8A). Increased apoptosis in sEng-expressing mice was supported by a significant increase in cleaved-caspase 3 by western blot analysis of whole retinas (Figure 8B and 8C).

**Figure 9:**
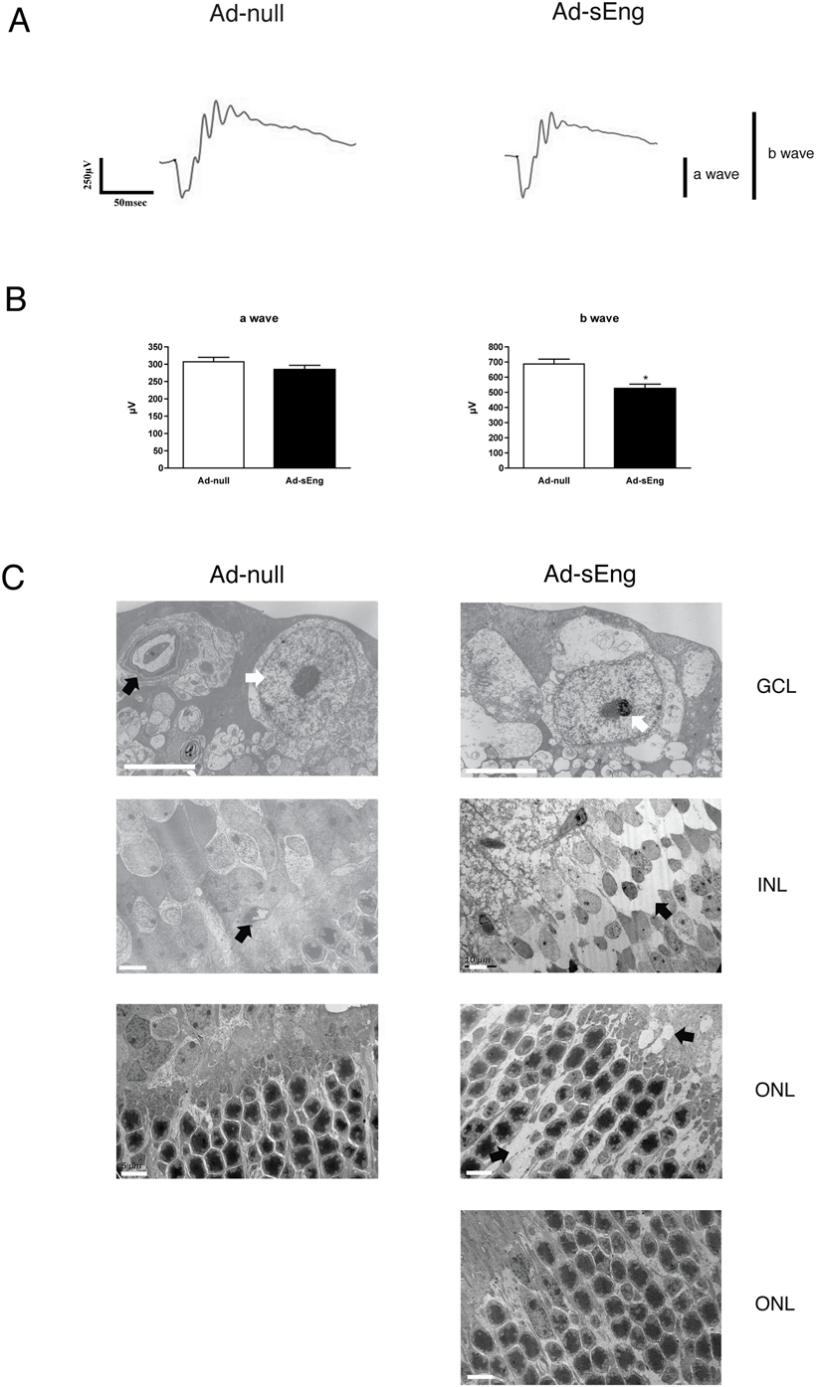
Inhibition of TGF-β leads to functional and morphological changes in retinal neural cells. (A) Following seven days of infection with Ad-null or Ad-sEng, retinal function was examined by scotopic ERG recording. (B) Inhibition of TGF-β did not alter the a-wave response, however b-wave was significantly decreased in Ad-sEng mice (n = 5, * p<0.05). (C) TEM of retinas from mice expressing Ad-null or Ad-sEng (day 14). Ganglion cells of sEng-expressing mice displayed features characteristic of apoptosis, with condensed nuclei (white arrow), cellular shrinkage and separation from surrounding extracellular matrix. Microvessels in Ad-null-infected mice appear normal in GCL and in the INL. Apoptosis was evident in the INL of sEng-expressing mice with separation of cells from surrounding tissue creating empty spaces (arrows) and apoptosis was apparent in the ONL of some sEng-expressing mice. Scale = 5 µm.

To determine if the reduced retinal perfusion in sEng-expressing mice was associated with retinal dysfunction, the electrical response of the retina to a light stimulus was measured via electroretinogram (ERG). Although the exact origin and generation of ERG waves remain incompletely understood, it is well accepted that the negative a-wave originates primarily from photoreceptor cells and the positive b-wave derives from the electrical response between photoreceptor cells and neural cells in the inner retina such as bipolar and amacrine cells [29]. Full-field ERG of Ad-null expressing control mice displayed baseline a- and b-wave amplitudes (Figure 9A). Analysis of sEng-expressing mice seven days after virus administration exhibited a-wave amplitude similar to Ad-null infected control, whereas the b-wave amplitude was significantly decreased (687.1+/−31.68 vs. 524.2+/−29.85) (Figure 9B).

Examination of the structural integrity of the neural retina by TEM revealed that TGF-β neutralization led to increased apoptosis of ganglion cells and neural cells of the INL and ONL (Figure 8C). Apoptotic Müller and amacrine cells were identified based on their morphology and location in the INL [30]. Ganglion cell, Müller cell and amacrine cell apoptosis in Ad-sEng mice was evidenced by condensed nuclei, cellular shrinkage and membrane blebbing. Photoreceptor cell nuclei displayed similar features of apoptosis and separation from the surrounding matrix in some, but not all mice. Axons beneath the ganglion cells were swollen, with apparent rupture of mitochondria.

### Inhibition of TGF-β1 signaling in EC - mesenchymal cell coculture leads to increased EC apoptosis

**Figure 10:**
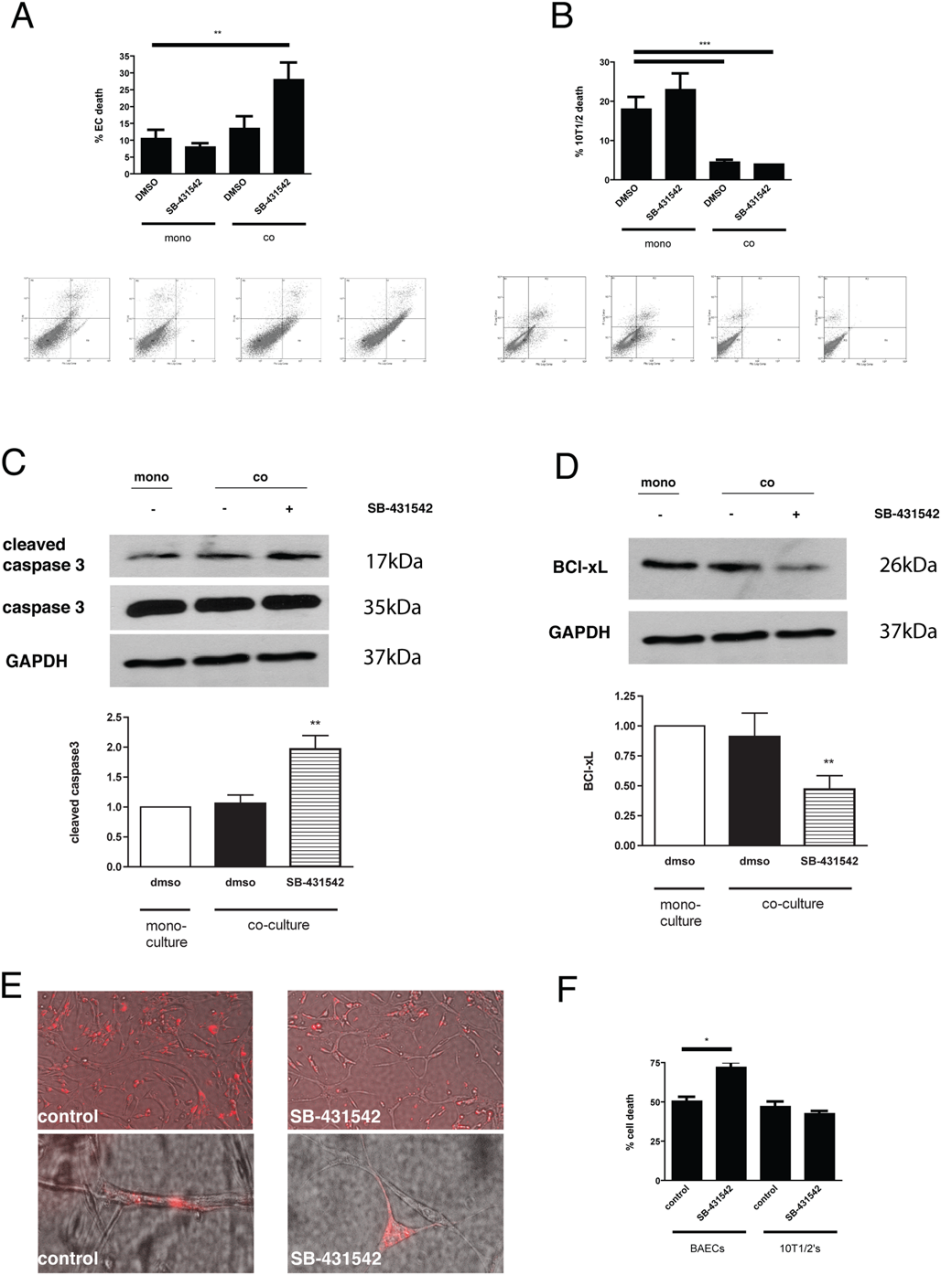
Inhibition of TGF-β signaling in EC-10T1/2 cell coculture leads to increased EC apoptosis. (A–B) Transwell cocultures of BAECs and 10T1/2 cells in the presence and absence of SB-431542, an inhibitor of ALK5. (A) Coculture of BAEC and 10T1/2 did not alter baseline levels of BAEC apoptosis. Addition of SB-431542 led to a significant increase in BAEC apoptosis in cocultures, but not monocultures. (B) Culture of 10T1/2 cells in the presence of BAECs significantly decreased baseline rates of 10T1/2 cell apoptosis. Addition of SB-431542 did not alter 10T1/2 cell apoptosis in either monoculture or in coculture with BAECs. Addition of SB-431542 increased cleavage of EC caspase 3 in co-culture with 10T1/2 cells (C) whereas the anti-apoptotic BCl-xl decreased (D). (E) Coculture of BAECs (unlabelled) in direct coculture with 10T1/2 cells (red) led to the formation of tube-like structures by EC, with 10T1/2 cells wrapped around and in close association with tubes. Addition of SB-431542 to BAEC-10T1/2 cocultures, after tubes had formed, led to the dissociation of 10T1/2 cells from the ECs and disassembly of tube-like structures. (F) Annexin V FACs assay for apoptosis of BAECs and 10T1/2 cells from (E) demonstrated a significant increase in EC apoptosis. Cells were retrieved using Matrigel dissociation solution and distinguished as red (10T1/2) or unlabeled (BAEC). Baseline apoptotic rates of both cell types in Matrigel are high as the cells that do not form tubes remain in the matrix and undergo apoptosis. Each experiment is representative of at least three independent experiments with similar results.

Since TGF-β1 acts on both EC and 10T1/2 cells via TGFβRI /ALK5 [2], we assessed the effects of inhibiting TGF-β1 signaling in Transwell cocultures. Whereas EC apoptosis was similar in mono-cultures of ECs and in EC-10T1/2 cell co-cultures, addition of SB-431542 increased endothelial apoptosis in the cocultures, but not in EC alone, suggesting that activated TGF-β1 is a survival signal for ECs (Figure 10A). Although 10T1/2 cell apoptosis was significantly decreased by coculture with EC; inhibition of TGF-β signaling with SB-431542 did not alter 10T1/2 cell apoptosis in the cocultures (Figure 10B), indicating that TGF-β1 does not mediate the increased mesenchymal cell survival observed in EC-10T1/2 cell cocultures.

In order to determine the mechanism of TGF-β1's anti-apoptotic effect on ECs, we examined cleavage of caspase 3, a key step in the apoptotic cascade, and the BCl-2 family of proteins, which also plays a role in apoptosis. Addition of SB-431542 increased cleavage of caspase 3 in ECs co-cultured with 10T1/2 cells (Figure 10C), whereas no change in cleavage of caspase 3 was detected between ECs from mono- or co-culture. In contrast, addition of SB-431542 decreased EC BCl-xl protein levels (Figure 10D).

To assess the role of TGF-β1 on EC-10T1/2 interactions in a three-dimensional coculture model, EC were cocultured with fluorescently pre-labeled 10T1/2 cells in Matrigel. EC formed tube-like structures, with the 10T1/2 cells associating with the endothelial abluminal surface and mimicking their association in vivo (Figure 10E). Addition of SB-431542 led to the dissociation of 10T1/2 cells from the EC, a reduction in the number of EC tubes (Figure 10D), and increased apoptosis of EC but not of 10T1/2 (Figure 10F).

## Discussion

Our findings reveal the existence of a constitutive TGF-β signaling in adult mouse retina required for the survival of both vascular and neural cells. Systemic inhibition of TGF-β led to numerous abnormalities in the retinal microcirculation, with impaired perfusion of the superficial vascular plexus and vascular leakage. These findings are consistent with the expression of TGF-β1 by pericytes and ECs of the human retinal microvasculature [23], the requirement for TGF-β1 and its receptors in formation of the vasculature [14], [15]. Further, these observations support our hypothesis that the relatively high ratio of pericytes to EC in the retina, compared to other vessel beds, should lead to significant activation of latent TGF-β1.

TEM analysis of retinas from sEng-expressing mice revealed collapsed vessels, consistent with an impaired capacity to form NO and to autoregulate flow. Coherent with this notion, NOS3, the enzyme required for constitutive NO formation in ECs has been shown to be downregulated in ECs harvested from HHT patients [31], a pathology due to heterozygous mutations in either endoglin or Alk1 and characterized by multiple vascular malformations. NO facilitates blood flow by acting upon the endothelium to maintain a non-thrombogenic surface. In addition, NO-induced contraction and relaxation of pericytes may regulate lumen size and thereby control capillary perfusion [32], functions supported by pericyte expression of the muscle isoforms of actin, myosin and tropomyosin [7]. As Pousielle's law dictates, minor changes in caliber of very small diameter capillaries would lead to significant alterations in blood flow. The high ratio of pericytes to ECs in the retina and brain may be of particular importance in regulating local blood flow because the microvasculature of the CNS lacks autonomic innervation and precapillary sphincters, which contribute to autoregulation in other microvascular beds [33].

During new vessel assembly, mesenchymal cells are recruited to forming capillary tubes via EC-derived PDGF-B, a process that has been demonstrated both in vitro and in vivo [3], [34]. Coculture studies have demonstrated that contact between these two cell types induces mesenchymal cell differentiation into mural cell phenotype and suppression of endothelial cell proliferation and migration - processes dependent upon TGF-β1 [2], [3], [35]. TGF-β1 has been shown to act on ECs via one of two type I TGF-β receptors (ALK1 & ALK5) to regulate cell fate decisions. ALK1 is activated in angiogenesis and its expression is diminished in adult vessels [36] whereas ALK5 mediates the anti-proliferative and anti-apoptotic actions of TGF-β1 signaling in ECs [37]. To understand the inhibitory effects of TGF-β1 in quiescent adult vasculature, ECs and mesenchymal cells were cocultured in Transwell or 3-D Matrigel in the presence of the ALK5 inhibitor SB-431542. Previous in vitro studies have revealed significant differences between TGF-β1 actions in conventional 2-D cell cultures and in 3-D matrices. In 3-D culture, TGF-β1 stimulates EC polarization, formation of zo-1 containing junctional complexes, redistribution and reorganization of the existing and newly synthesized ECM; none of these effects are seen in parallel 2-D in vitro experiments [38]. For these reasons we compared the effect of TGF-β1 inhibition on EC and 10T1/2 cell apoptosis in 2-D Transwell or 3-D cocultures. In both coculture systems, inhibition of ALK5 led to a significant increase in endothelial, but not 10T1/2, cell death. Analysis of EC samples from Transwell revealed an increase in cleavage of caspase 3, concomitant with a decrease in the anti-apoptotic BCl-xL upon TGF-β1 inhibition. Consistent with these findings, BCl-xL has previously been described as a negative regulator of caspase 3 [39]. Previous 2-D coculture studies found that 10T1/2 cell-derived VEGF inhibits EC apoptosis [40], however addition of TGF-β1 can also induce apoptosis in the absence of supporting cells, demonstrating the effects of TGF-β1 are context-dependent [41]. Our observation that the presence of 10T1/2 cells did not significantly alter endothelial apoptosis may be due to the higher levels of serum and/or culture in Matrigel, which both contain VEGF. Furthermore, ALK5 inhibition led to a significant decrease in the number of mesenchymal cells associated with ECs in the 3-dimensional model, suggesting that TGF-β1 is required for the continued association between these cells. Similarly, we have showed previously that addition of neutralizing agents (soluble TGF-βRII) blocks cord formation and 10T1/2 differentiation [20]. Activated TGF-β1 blocks cell migration and leads to changes in matrix level and composition [42] [4]; both are likely to contribute to the observed decrease in cell association in SB-431542-treated cocultures. Our findings are consistent with observations of background diabetic microangiopathy in which loss of vessel-associated pericytes is thought to underlie capillary dilation as well as breakdown of the BRB and also a decrease in active TGF-β1 in DR [43].

Our studies demonstrate perfusion abnormalities in sEng-expressing mice. Results of fluorescein angiography, Evans blue extravasation, and western blotting for association of the tight junction proteins occludin and zo-1 suggest that endogenous TGF-β1 is required to maintain the BRB. In concordance with these observations in vivo, coculture of ECs and 10T1/2 cells led to enhanced EC barrier function, and the addition of the ALK5 inhibitor reversed this effect and reduced coculture-induced association between occludin and zo-1. Furthermore, previous reports demonstrate that systemic administration of a TGF-βR1 inhibitor (Calbiochem: LY364947) increases tumor vessel permeability [44] and smad4 deficient mice exhibit defects in vascular integrity and maturation as well as abnormal gap junction formation [45]. Furthermore, the finding that permeability defects in Akt(−/−) mice can be rescued with addition of TSP-1 and TSP-2, both potent activators of TGF-β1 in vivo [46], further support a role for TGF-β1 in the maintenance of the BRB. These results suggest that locally activated TGF-β1 is critical for optimal endothelial barrier function and are in agreement with our previous reports demonstrating that TGF-β1 induced by astrocytes contacting ECs contributes to the maintenance of the blood-neural-barrier [47].

While there was no change in the levels of the tight junction proteins zo-1 and occludin in retinas from sEng-expressing mice (data not shown), there was a decrease in association between these proteins, a characteristic feature of tight junction disassembly [28]. Similarly, the coculture-induced association between occludin and zo-1 was reversed with addition of the ALK-5 inhibitor. Integrity of inter-endothelial junctions is regulated by multiple parameters including cytoskeletal tension, junctional protein-protein interaction and connection between junctional proteins and the actin cytoskeleton. Each of these determine the intercellular cleft size and degree of permeability. For example, binding of zo-1 to the COOH-terminal cytoplasmic tail of occludin plays a role in maintaining the tight junction and also cytoskeletal tethering [48], whereas previous studies describe the association of occludin/zo-1 as dependent upon both tyrosine phosphatase signaling and also protein kinase C [49].

High levels of circulating sEng also led to significant neural cell apoptosis, particularly of the ganglion cells and neural cells of the INL. The pattern of apoptotic cells correlated well with the localization of pp-smad2, the direct downstream target of TGF-β1, suggesting that the apoptosis and functional defects of neural retinal cells are the direct effect of a role for TGF-β1 or TGF-β3 as a survival factor. In support of this notion, TGF-β1 has been described in the monkey and human ganglion cells of the retina, the outer plexiform layer (OPL), the axon bundles in the nerve fiber layer; and the associated ganglion cell bodies proximal to the basal lamina comprising the inner limiting membrane [50]. The TGF-β1 precursor molecule LC pre-pro is evident in the photoreceptor outer segments, the OPL and Muller cell endfeet cytoplasm. In normal adult rat retinas, both protein and mRNA of TGF-βRI and TGF-βRII are present in the ganglion cells [51]. TGF-β3 is not associated with microvascular cells, but is present in neural cells of the ganglion cell layer, the inner nuclear layer and some photoreceptor cells. The expression of TGF-receptors and pp-smad2 in normal retinas suggests that TGF-β plays an important role in the homeostasis of normal retina [52], with effects independent of the vasculature. These findings of impaired retinal function with inhibition of TGF-β have important implications for understanding both vasoproliferative and vascular degenerative retinal diseases.

## Materials and Methods

### RNA isolation and PCR

Retinas were dissected, and pooled for RNA extraction using a RNAqueous - 4PCR kit (Ambion). RNA was reverse-transcribed using Superscript II reverse transcriptase (Invitrogen). Standard PCR was performed with 1 U Taq DNA polymerase (Roche Diagnostics) and 0.2 mM of appropriate primer pair for 30 cycles. Genes monitored were smad2, smad3, TGF-β1, TGF-β3 and glyceraldehyde phosphate dehydrogenase (GAPDH). Primer pairs (Invitrogen) used were smad2 (For: CGGAGATTCTAACAGAACTG; Rev: TGCTTGAGCATCGCACTGAA), smad3 (For: AGCACACAATAACTTGGACC; Rev: TAAGACACACTGGAACAGCGGATG), TGF-β1 (For: GCTGCGCTTGCAGAGATTAAA; Rev: TTGCTGTACTGTGTGTCCAG), TGF-β3 (For: GCTCTTCCAGATACTTCGAC; Rev: AGCAGTTCTCCTCCAGGTTG) and GAPDH (For: GTGGCAAAGTGGAGATGGTTGCC; Rev: GATGATGACCCGTTTGGCTCC). Following PCR, reaction products were analyzed by agarose gel electrophoresis on 2.5% gels with 100 bp ladders as size standards, and visualization by ethidium bromide staining.

### Animals

All animal protocols were approved by the Schepens Eye Research Institute IACUC and mice were handled in accordance with the ARVO statement for the Use of Animals in Ophthalmic and Vision Research. For in vivo neutralization of TGF-β, adult CD-1 mice were injected via tail vein with 1×10^∧10^ viral particles (VP) Ad-CMV-null (Ad-null) or 2.5×10^∧9^ VP Ad-CMV-sEng (Ad-sEng) (Q Biogene, Montreal, Canada) with the day of injection considered as day zero. Blood was collected in EDTA tubes on day seven by submandibular vein or by cardiac puncture on day 14 and plasma was collected and stored at −80°C. Plasma sEng, measured via ELISA (R&D Systems, Minneapolis, MN), was approximately 200 ng/ml seven days following injection.

### SDS-PAGE and immunoblot analysis

Cell pellets were treated with lysis buffer and equal protein was fractionated by 10% (wt/vol) polyacrylamide resolving gels. After transfer to nitrocellulose membranes, non-specific protein binding was blocked by a 60-min incubation in PBS-T (phosphate-buffered saline, 0.1% Tween-20) containing 5% (wt/vol) nonfat skim milk. Membranes were then incubated overnight at 4°C with either pp-smad2 (1∶100; Chemicon, Temecula, CA) or caspase 3 (1∶100; Cell Signaling) antibodies diluted in PBS-T with 2.5% BSA. After two 10-min washes with PBS-T, membranes were incubated with horseradish peroxidase–conjugated rabbit polyclonal IgG antibody (1∶300) for 90 min at room temperature. After two further washes with PBS-T, immunoreactive proteins were identified by enhanced chemiluminescence. Scanning densitometry was performed with image-analysis software (ImageJ).

Changes in occludin/zo-1 co-association were monitored by immunoprecipitation, performed as previously described [53] with minor modifications. Cells lysates (50 µg) were incubated with 1.75 µg of anti-occludin antibody (final volume of 500 µl) and incubated overnight at 4°C with continuous rotation. Complexes were captured with 50 µl protein A beads (50% slurry; Upstate), washed in PBS, resuspended in 25 µl of SDS-PAGE sample buffer and heated for 5 min at 90°C. Beads were pelleted and supernatant examined by Western blotting as described above with anti-zo-1 antibodies (1∶500; Zymed).

### Immunohistochemistry

Cryosections were blocked with 0.2% Tween, 3% donkey serum and 3% goat serum in PBS (Sigma, St Louis, MO), then incubated overnight at 4°C with primary antibodies diluted in blocking solution (pp-smad2, 1∶500; Chemicon, Temecula, CA). Secondary antibody of biotin-conjugated goat anti-rabbit (Vector Labs, Burlingame, CA) were added, and were visualized with avidin-biotin-peroxidase technique and 3, 3′-diaminobenzidine (DAB) substrates (Vector ABC kit). Each experiment included a section incubated with isotope-matched IgG as a negative control. Images were captured with a Zeiss Axioskop 2 MOT plus microscope.

### Fluorescein perfusion

Following 14 days of infection, mice were perfused with seven ml of fluorescein dextran 2×10^∧6^ m.w. (Sigma, St Louis, MO) (50 mg/ml in 4% paraformaldehyde) in PBS pre-warmed to 37°C. Perfusion was accomplished from a 21-gauge cannula inserted into the aorta via the left ventricle, allowing blood and fixative to exit via an opening in the right atrium. Tissues were removed, fixed in 4% paraformaldehyde at 4°C overnight and processed for cryosections or retinas were isolated and flat-mounted for visualization by confocal fluorescence microscopy using a Leica TCS Sp2 confocal microscope. Composites were generated using Adobe Photoshop.

### Quantification of vascular perfusion

Cryosections (10 µm) of retinas from mice perfused with FITC-dextran were blocked overnight at 4°C (in PBS, 0.2% Tween, 3% donkey serum and 3% goat serum), then incubated at 4°C overnight with rabbit anti-collagen type IV polyclonal (1∶400; Abcam, Cambridge, MA) followed by a rhodamine-conjugated anti-rabbit secondary antibody (1∶300; Jackson Immunoresearch, West Grove, PA). Perfusion of retinal vessels in the ganglion cell layer was measured by comparing the number of collagen IV-positive vessels to the number of FITC positive vessels on successive cryosections. For each animal, three cryosections separated by 150 µm were quantified.

### Blood flow autoregulation

To assess peripheral autoregulation capacity, flow rates (µl/cycle) were measured in the tail using a CODA 6 non-invasive blood pressure system (Kent Scientific, Torrington, CT) under 2% isoflurane anesthesia. Seven days post injection, baseline flow rates were established in Ad-null or Ad-sEng mice, before measuring vasoactive response to retro-orbital injection of 12.5 µg/kg acetylcholine (Ach) (total volume 100 µl), an endothelium dependent vasodilator.

### Fundus angiography

Seven days after adenovirus injection, mice were anesthetized and their pupils dilated with 1% atropine sulfate. Fluorescein angiography was performed after intraperitoneal injection of 0.05 ml of 25% fluorescein sodium (Akron). Photographs were taken with a preset 20D lens appositioned to the fundus camera lens at regular time (from 1 min to 4 min post I.P injection).

### Evans blue permeability

Vascular permeability was assessed using Evans Blue as previously described [17]. Seven days after injection, mice were anesthetized and injected via tail vein with 2% Evans Blue dye. After 40 min mice were perfused via heart puncture with PBS containing 2 mM EDTA for 20 min. Retinas were harvested and incubated in formamide and rotated at 70°C for 24 hr. Supernatants containing extravascular Evans blue were collected after centrifugation at 14,000 rpm for 10 min. The optical density (OD) was measured at 620 nm. The following formula was used to correct OD for contamination with heme pigments: OD620 (corrected) = OD620−(1.326×OD740+0.030).

### Transmission electron microscopy (TEM)

Following 14 days of adenoviral infection, mice were perfused with fluorescein as described above except that 10 ml sodium cacodylate buffer 0.2 M, pH 7.4, followed by 10 ml of half strength Karnovsky's fixative was used. Retinas were dissected and fixed with half strength Karnovsky's fixative, followed by 2% osmium tetroxide and en block stain with 0.5% uranyl acetate. After dehydration and embedding, ultra-thin sections were visualized using a Phillips 410 transmission electron microscope.

### Scanning electron microscopy (SEM)

Transwell cultures were washed in PBS and fixed in half-strength Karnovsky fixative, rinsed in PBS and dehydrated in a graded series of ethanol solutions. After drying in a critical point dryer (Samdri-795; Tousimis, Rockville, MD), samples were coated with a 150A carbon layer (Ion Beam Coater; Gatan, Pleasanton, CA). Cell images were captured on a scanning electron microscope (FESEM 7401F; Field-Emission Scanning Electron Microscope; JEOL, Peabody, MA) using backscatter detection to visualize the gold labeling.

### Cell culture

For cocultures, 10T1/2 cells were cultured on the underside of Transwell inserts (0.4 µm pore size, Co-star) for four days at confluence before addition of BAECs and culture for a further three days in DMEM 10% FBS. The TGF-β signaling inhibitor, SB-431542 (Tocris) (10 µM), which inhibits ALK5, but not ALK1, kinase activity, [54] was added in DMEM containing 1% FBS for 24 hr. Control wells contained equivalent volumes of DMSO.

For 3 dimensional cocultures, 8.0×10^4^ BAECs and 2.0×10^4^ 10T1/2 cells were cocultured for five days in 400 ul of 50% Matrigel™(BD Biosciences) in 10% FBS, 40% DMEM, 10 ng/ml VEGF (obtained from the NIH-NCI Preclinical Repository). Media containing 10% FBS overlaid each gel. To distinguish between the two cell types, 10T1/2 cells were pre-labeled with PKH26 red fluorescent linker dye (Sigma, St Louis, MO). To examine effects of ALK5 inhibition, 10 µM SB-431542 was added for 72 hr in DMEM containing 1% FBS. Control gels were treated with equivalent amounts of DMSO. Apoptotic cells were detected using the Vybrant® Apoptosis Assay Kit (Invitrogen, Carlsbad, CA) followed by FACS analysis using a FACSCAN flow cytometer.[55] Cells were retrieved from Matrigel with BD cell recovery solution (BD Biosciences) as per the manufacturer's instructions.

### Transendothelial permeability assay

BAECs-10T1/2's were grown on Transwell filters in the presence or absence of SB-431542 (10 µM). FITC-dextran (0.5 mg/ml; 40 kDa; Sigma, St Louis, MO) was added to the upper chamber. Aliquots (100 µl) were collected over time from the lower chamber (5, 15, 30, 60, 120, 240 min). The liquid volume and hydrostatic pressure in the lower chamber were maintained by replenishing 100 ul of media (+/−SB-431542) to the lower chamber at each time point. Fluorescence measurements were determined immediately using a fluorescence reader (excitation maximum 490 nm; emission maximum: 520 nm).

### Electroretinogram recording (ERG)

Retinal function was assessed using a UTAS-E3000 recording system (LKC, Technologies, Inc. Gaithersburg, MD). Mice were dark-adapted for 6 hr and anesthetized with a mixture of ketamine/xylazine (120 mg/kg and 10 mg/kg, respectively). Pupils were dilated with drops of 1% tropicamide and 1.5% cyclopentolate. The active electrode was a gold wire loop on the cornea, the reference electrode was placed in the head, and the ground electrode was placed in the back. Each mouse was placed in front of a Ganzfeld bowl (UTAS3000; LKC Technologies) that presented a series of flashes with increasing intensity. ERG response to a series of increasing intensity light flashes: +10-dB was averaged over 10 separate flashes per light intensity. The inter-stimulus interval was 1 minute for all flash intensities. The a-wave amplitude was measured from the baseline to the trough of the first negative wave and the b-wave amplitude was measured from the trough of the a-wave to the peak of the positive wave or when the a-wave was not present, from baseline to the peak of the positive wave.

### TUNEL assay

Apoptotic cells were detected using the Promega Dead End HRP kit (Promega, Madison, WI), following the manufacturer's procedure with some modifications. Sections were permeabilized for 5 min in cold PBS containing 0.2% Tween-20, then pre-equilibrated with equilibration buffer and incubated at 37°C in TUNEL reaction mix containing biotinylated nucleotides and TdT (terminal deoxynucleotidyl transferase) enzyme. The reaction was terminated after 1 hr with 2× SSC solution for 15 min at RT. Biotinylated nucleotides were detected by incubation with Strepavidine-HRP followed by DAB incubation. Staining was stopped by washing in PBS and sections were lightly counterstained with hematoxylin prior to mounting.

### Statistical analysis

In all experiments, unless otherwise indicated, data are reported as mean±SD in at least 3 replicates per group. Data were analyzed by student's unpaired T-Test. P values <0.05 were taken to indicate statistical significance.
